# Care of the Newborn

**Published:** 1914-07

**Authors:** 


**Care of the Newborn.** Leo-Wolf in Archives of Pediatrics, May, 1914, urges careful regulation of the feeding of newborn, and is an adherent of the four hour intervals. In the hospitals the newborn should be placed under the pediatric service from the first. The bath and the handling of the newborn should be done aseptically, and the mouth should never be wiped out. A new and modern book on the care of infants is needed.

